# Evaluation of a community-based remotely delivered exercise program (2Unstoppable Strong) for women diagnosed with cancer

**DOI:** 10.1007/s00520-025-09772-4

**Published:** 2025-07-25

**Authors:** Angela J. Fong, Isabel L. Wakefield, Betoul Ali, Ilana Gamerman, Stacey Mann

**Affiliations:** 1 School of Kinesiology, University of Michigan, Ann Arbor, USA; 2 School of Public Health, University of Michigan, Ann Arbor, USA; 3 Fairfax, USA

**Keywords:** Physical activity, Exercise, Remote-delivered, Cancer survivor, Health-related quality of life, Community-based

## Abstract

**Purpose:**

Few remote-delivered physical activity (PA) interventions leverage a community-academic partnership, which may impact sustainability. The current study aimed to assess the satisfaction  with a community-based remote-delivered group-based PA program (2Unstoppable Strong) among women diagnosed with cancer and the preliminary impact of the intervention on exploratory outcomes (PA levels, perceived social support, exercise self-efficacy, barrier self-efficacy, and health-related quality of life).

**Methods:**

Online questionnaires were administered at baseline (T0), post-program (T1), and follow-up (T2). Quantitative analyses included descriptive statistics and repeated measures analyses of variance. Open responses at the end of the questionnaire were used to identify areas of improvement. Content analysis was used to analyze the open response data.

**Results:**

Participants rated 2Unstoppable Strong as satisfactory (*M*_score_ = 4.7, *SD* = 0.54; *M*_range_ = 2.93 to 5.00). Moderate-to-vigorous physical activity significantly increased from T0 to T1 to T2 (*F* (2, 34) = 3.5, *p* < .05). From T0 to T1 to T2, both perceived social support quantity (*F* (2, 34) = 18.6,* p* < .001) and quality (*F* (2, 34) = 13.9, *p* < .001) significantly increased. All exploratory outcomes did not significantly change. Participants enjoyed interacting with the trustworthy instructors and found the videoconferencing software convenient to use. Areas for improvement included facilitating social interaction within the exercise group, increasing the intervention duration, and providing additional sessions.

**Conclusions:**

Based on the promising findings, future hybrid implementation-effectiveness studies with larger and more-diverse populations are needed to examine the impact of 2Unstoppable Strong on cancer-related outcomes.

**Supplementary Information:**

The online version contains supplementary material available at 10.1007/s00520-025-09772-4.

## Introduction

In the USA, the 5-year relative survival rate for all cancers combined is 69% for those diagnosed between 2014 and 2020 [1]. Despite these advancements, cancer survivors, defined as the time of treatment onwards, experience deleterious long-term and late physical and psychosocial effects because of the cancer diagnosis and treatment [2]. Physical effects such as pain, lymphedema, and impaired physical function in conjunction with psychosocial effects such as increased cancer-related fatigue and anxiety are commonly associated with lower health-related quality of life (HRQoL) [3]. Consequently, it is essential to develop effective strategies to mitigate the long-term and late effects and improve HRQoL among cancer survivors including those with metastatic disease.

Physical activity (PA) is one such strategy which has demonstrated numerous physical and psychosocial benefits among cancer and metastatic cancer survivors and positively impacts HRQoL [4]. Based on robust evidence, PA improves physical fitness, physical function, and psychosocial well-being including self-efficacy and HRQoL [5]. Importantly, PA is safe and well tolerated after cancer treatment [6]. Cancer survivors enrolled in a combination of aerobic and resistance exercise interventions had a significantly decreased risk of all-cause mortality and cancer recurrence when compared to control groups not receiving any major exercise intervention [7]. As a result, the American College of Sports Medicine (ACSM) recommends that cancer survivors engage in 150 min/week of moderate-to-vigorous physical activity (MVPA) with at least two sessions of resistance exercise [4, 6]. However, and estimated 34% to 47% of cancer survivors are meeting ACSM guidelines for aerobic, resistance, or combined aerobic and resistance exercise [4, 8]. As a result, efficacious PA interventions are needed to increase PA levels among cancer and metastatic cancer survivors.

Low levels of PA among cancer survivors can be attributed to many barriers. Low self-efficacy for exercise and low self-efficacy for overcoming common barriers to exercise, such as exercising when the weather is bad, are determinants of low PA levels [9]. Moreover, barriers such as lack of time and transportation costs hinder regular PA [10]. Specifically, many supervised PA interventions require individuals to meet in-person at a location outside of the home, which exacerbates access-related barriers [11]. As a result, PA interventions developed and implemented for cancer survivors that increase exercise-related self-efficacy and mitigate access-related barriers may positively impact PA levels [12].

Remote-delivered PA interventions increase accessibility and mitigate commonly reported barriers to PA among cancer survivors [13] and metastatic cancer survivors [14]. Previous remote-delivered PA interventions have demonstrated high feasibility and acceptability among cancer survivors [15]. Furthermore, there is increasing evidence that remote-delivered PA interventions are associated with increased mean daily light PA levels, improved physical function, and higher HRQoL [14, 16–19]. However, participants commonly report the lack of social support and lack of interaction as negative components [12, 16]. Therefore, it is imperative to facilitate social support in remote-delivered interventions to facilitate increasing cancer survivors’ PA levels [20].

While the growing evidence of the positive psychosocial impacts of remote-delivered PA interventions is encouraging, the sustainability is unclear [18]. Leveraging a community-academic partnership, which balances scientific rigor and pragmatism, has the potential for PA intervention sustainability [21]. The current research is a community-academic partnership between a non-profit organization, 2Unstoppable, based in Fairfax, VA, and a large public university, the University of Michigan, based in Ann Arbor, MI. The mission of the non-profit organization is to help women diagnosed with cancer become more active and develop social support. The founders of the non-profit organization developed and implemented a remote-delivered PA program called 2Unstoppable Strong (2U Strong). Briefly, 2U Strong is a supervised, remote delivered PA program that leverages social support, focuses on aerobic and muscular fitness, flexibility, and balance (refer to the “Methods” section for details). While 2U Strong has received positive testimonials from previous participants (https://2unstoppable.org/2unstoppable-strong/), ratings of satisfaction and the impact of the program on social support and PA levels are not as clear.

Guided by community-based research principles [22], members of the non-profit organization are treated as co-investigators (IG and SM) by guiding the research question and evaluation process. The current study aimed to (a) assess participants’ overall satisfaction rating of 2U Strong (primary outcome) compared to a predetermined benchmark (*M*_score_ > 4.0 out of 5); (b) evaluate the preliminary impact of the intervention on exploratory outcomes (PA levels, perceived social support, exercise self-efficacy, barrier self-efficacy, and HRQoL; and (c) qualitatively identify areas of improvement for the 2U Strong program.

## Methods

### Study design

The research used a single-arm longitudinal study design using online questionnaires and open-ended text responses. Institutional review board approval was obtained from the original host institution (Rutgers Cancer Institute) to conduct the evaluation research (IRB# Pro2021002042) and a data user agreement (23- UFA04108) with the University of Michigan following the lead author’s (AJF) move to analyze the deidentified data. Given the community-academic partnership, responsibilities were separated between non-profit organization and research staff. The non-profit organization staff were responsible for recruiting and enrolling women diagnosed with cancer into 2U Strong and conducting the exercise program. Women diagnosed with cancer including those with metastatic disease learned about 2U Strong through advertisements in community and clinical settings such as community centers, gyms, and hospitals, word-of-mouth, social media, and local newspapers. Those who were interested in increasing their exercise levels were screened for eligibility using videoconference software by non-profit organization staff. Reasons for ineligibility included inability to exercise independently and scheduling conflicts. Following 2U Strong enrollment but prior to program start, enrollees were informed about the voluntary evaluation research component. The names and contact information of interested enrollees were given to the research staff at the host institution. The research staff were responsible for all program evaluation-related procedures including screening enrolled 2U Strong participants for interest in the research over the phone or online, informed consent, questionnaire delivery, and incentive distribution.

### Participants and procedures

2U Strong enrollees (*N* = 63) were informed about the opportunity to engage in the evaluation research component by non-profit organization staff. A research recruitment flyer and e-mail script were given to non-profit organization staff to ensure consistency and accuracy of information provided to enrollees. Interested enrollees provided contact information to non-profit organization staff who then emailed the information to research staff. Research staff confirmed study interest via email or phone and screened enrollees for eligibility. Women enrolled in 2U Strong were eligible for the evaluation research component if they (a) ≥ 18 years old; (b) self-reported a cancer diagnosis; (c) completed active treatment ≥ 6 months to 5 years prior to exercise program commencement (hormonal- and immunotherapies were acceptable) or self-reported metastatic disease; (d) completed 2U Strong screening assessment with non-profit organization staff; and (e) were able to read and understand English. Women were excluded if they met any of the following criteria: (a) respiratory, joint, or cardiovascular problems precluding physical activity; and/or (b) planned elective surgery during the duration of the study period.

**Fig. 1:**
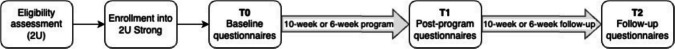
Flow diagram of 2Unstoppable strong pilot study

REDCap was used to collect data at T0 (i.e., baseline or 1–2 weeks prior to program start), T1 (i.e., post-program or 1–2 weeks after program end), and T2 (i.e., follow-up or either 10- or 6-week follow-up; Fig. 1). Participants received a $25 e-gift card for each questionnaire completed where completion was defined as 80% of items contained a response. Data were collected from January 2022 to August 2023.

#### Exercise program (2U Strong)

The non-profit organization, as guided by a personal trainer and group fitness instructor with certification in exercise and cancer, developed and implemented a remote, community-based exercise program. This free program was 10 weeks for women diagnosed with cancer and 6 weeks for women diagnosed with metastatic cancer. A shorter program duration was selected for metastatic cancer survivors to decrease the likelihood of dropout [23]. The exercise program was group-based with 6 to 10 participants and one instructor or 4 to 6 participants with metastatic cancer and one instructor and delivered over Zoom. Instructors were group fitness instructors with certification in exercise and cancer. Each participant was assigned a social support partner (also called a 2U Buddy) who was a member of the same exercise group. The exercise program is evidence-informed based on the cancer-specific exercise guidelines [4, 6]. A week-by-week description of the exercise program can be found in Supplemental Files (Supplemental Table 1), which targeted aerobic and muscular fitness, flexibility, and balance. Participants aimed for a moderate intensity based on if they could hold a conversation while exercising. Exercises were modified as needed for all participants. Participants were expected to attend one supervised group-based session (45 min in duration) per week and complete two unsupervised individual sessions on their own. On a weekly basis, participants were emailed a link to the session recording to guide the unsupervised exercise sessions. All participants were mailed resistance bands.

### Measures

#### Participant characteristics

Self-reported sociodemographic data included age (years), race (White, Black or African American, Asian or Asian American, or Other [including American Indian or Alaska Native, Native Hawaiian or other Pacific Islander, and multiracial]), and Hispanic ethnicity (yes or no), US nativity (yes or no), current marital status (married, divorced, separated, or single/never married), education (high school graduate or equivalent, post-secondary, some college with no degree, associate degree, Bachelor’s degree, and graduate or similar), and annual household income.

Given the community-based study design, a pragmatic approach was used to collect cancer- and health-related data, which were also self-reported. Variables included cancer diagnosis (open text response), cancer diagnosis stage (I through IV), treatments received (chemotherapy, radiation, surgery, other), and menopausal status.

#### Satisfaction (primary outcome)

Satisfaction was measured using a modified version of the Internet Evaluation and Utility Questionnaire, which assesses participants’ experience and perceptions of Internet-based interventions for health behavior change. The questionnaire has 14 items where participants respond on a 5-point Likert scale anchored from 1 = “not at all” to 5 = “very.” Eight constructs are measured including ease of use; convenience; engagement; enjoyment; privacy; satisfaction; and acceptability. The questionnaire has been validated among a sample of cancer survivors with insomnia (Cronbach’s alpha = 0.69) [24]. The modified version used did not include the layout construct because it was not applicable, and the adapted version was not tested prior to use in this study.

#### Physical activity levels (exploratory outcome)

Physical activity levels were assessed through self-report using a modified version of the Godin Leisure Time Exercise Questionnaire [25]. Participants self-report the number of sessions within the previous 7 days during which they engaged in at least 30 min of mild, moderate, and vigorous activity and the average duration. For the purposes of this investigation, total minutes spent in the different physical activity intensities were calculated by multiplying the number of sessions by the average duration. Then, minutes of weekly physical activity were calculated for the moderate-to-vigorous intensities and for total physical activity including mild, moderate, and vigorous intensities. This measure has demonstrated acceptable reliability and validity among cancer survivor populations [26].

#### Perceived social support (exploratory outcome)

An adapted version of the Social Support Survey was used to assess social support for exercise using a total of seven items [27]. Participants rated satisfaction with current quantity and quality of social support for exercise and quality of social support for exercise using two items. Items are rated on a 5-point Likert scale where 1 = “very dissatisfied” and 5 = “very satisfied.” Next, participants completed a short checklist of sources of support for exercise (e.g., no one, friend with cancer, family, online support groups). To understand the satisfaction with perceived social support received from their assigned 2U Buddy, participants rated the amount their exercise partner supports their exercise efforts based on different types of social support including listening support (i.e., people who listen to you without giving advice or being judgmental); emotional support (i.e., people who comfort you and indicate to you that they are on your side); reality confirmation (i.e., people who are similar to you, who help you confirm your perceptions and perspectives of the world and help you keep things in focus); and tangible assistance (i.e., people who offer you transportation, financial assistance, gifts, and/or products) using four items. The measured was scored as four separate scales: perceived social support quantity, perceived social support quality, sources of social support, and satisfaction with 2U Buddy support (mean score) to assess different aspects of social support and sources of support [28].

#### Exercise self-efficacy (exploratory outcome)

The Exercise Self-Efficacy Scale is an 8-item scale assessing confidence in one’s ability to exercise at a moderate intensity three times per week [29]. The scale is anchored at 0% = not at all confident to 100% = complete confidence.

#### Barrier self-efficacy (exploratory outcome)

The Barrier Self-Efficacy Scale has 13 items and is designed to understand subjects’ perceived capabilities to exercise three times per week over the next 3 months in the face of commonly reported barriers such as bad weather, boredom, and going on a vacation. The scale is anchored at 0% = not at all confident to 100% = complete confidence [30].

#### Health-related qualify of life (exploratory outcome)

The Short Form-12 assessed domains of health-related quality of life including Physical health-related domains include General Health, Physical Functioning, Role Physical, and Body Pain [31]. Mental health-related scales include Vitality, Social Functioning, Role Emotional, and Mental Health. The scores range from 0 to 26 with higher scores indicating better HRQoL [32].

#### Program feedback (exploratory outcome)

Feedback was gathered through open-ended questions at T1. Participants were asked to list three aspects of the program they liked, three aspects they did not like, and to provide suggestions for improvement.

### Data analysis

Quantitative data was summarized using descriptive statistics including means, standard deviations, and percentages. A visual inspection used to assess if mean satisfaction scores exceeded the predetermined benchmark, defined as if the mean participant rating of the program was a 4.0 out of 5. Repeated measures analyses of variance (ANOVAs) were conducted to examine the main effect of time (T0 (baseline) to T1 (post-program) to T2 (follow-up)) on the exploratory outcomes. All tests were two-sided and significant at *p* < 0.05. Assumptions of sphericity were not violated (all *ps* > 0.05). The effect sizes (${\eta }_{p}^{2}$) of the exploratory outcomes were reported regardless of the statistical significances, where a small effect is 0.01, a medium effect is 0.06, and a large effect is 0.14 [33]. Missing data were determined to be missing completely at random (MCAR) based on Littles’ MCAR test (*p* > 0.05) and were handled using listwise deletion [34, 35]. All analyses were conducted with SPSS version 29 (Armonk, NY).

The open-ended feedback responses were coded using content analysis to identify commonly reported positive feedback and areas for improvement [36]. Participants’ responses were organized inductively into themes based on frequency counts. Two authors (AJF and BA) conducted the qualitative analysis without software.

## Results

### Descriptive results

Out of 63 women, 54 completed the 2U Strong program during the study period from January 2022 to August 2023 (85.7% completion rate). Out of 63 women who enrolled in 2U Strong during the study period, 44 agreed to be enrolled in the research (70.0% acceptance rate). Of the 44 participants, *n* = 3 did not complete the T1 questionnaires and *n* = 4 did not complete T2 questionnaires. Only one participant was considered a dropout at T0 because they did not complete both T1 and T2 questionnaires. Missing data (cases) were removed from the dataset and the final analytical sample was *n* = 36 participants (81.8% retained from those who agreed to the research).

At T0, participants (*N* = 44) were an average age of 53.9 (*SD* = 53.0) years old, self-identified as mostly White (81.4%) and non-Hispanic (97.7%), were married (58.1%), and had an annual household income of $100,000 or more (37.2%). Participants commonly self-reported being diagnosed with breast cancer (88.4%) and more than a third were stage IV or metastatic cancer survivors (37.2%). On an average week, participants reported engaging in 71.8 min (*SD* = 60.0) of total physical activity across light, moderate, and vigorous intensities. However, participants reported engaging in 30.9 min (*SD* = 31.0) of moderate-to-vigorous PA, which is below the recommended amount of 150 min per week. Participants were “somewhat satisfied” with their quantity (*M* = 2.79 out of 5, *SD* = 1.2) and quality (*M* = 2.86 out of 5, *SD* = 1.3) of social support. Commonly reported sources of social support were family member without cancer (44.2%), no one (34.9%), and a friend without cancer (20.9%). A summary of the T0 participant characteristics can be found in Table 1.

**Table 1:** Summary of baseline characteristics among participants (*N* = 44)

Characteristic	*M (SD)* or *n* (%)
Age (years)	53.9 (53.0)
Race White Black or African American Asian or Asian American or Pacific Islander Other or multiracial	35 (81.4%) 6 (14.0%) 1 (2.3%) 1 (2.3%)
Ethnicity Non-Hispanic Hispanic	42 (97.7%) 1 (2.3%)
US nativity Yes No	40 (93.0%) 3 (7.0%)
Marital status Married Divorced/single or never been married	25 (58.1%) 18 (41.8%)
Education Some college, but no degree Associate degree Bachelor’s degree Graduate degree or similar	2 (4.7%) 2 (4.7%) 9 (20.9%) 30 (69.8%)
Household income $25,000 to $49,999 $50,000 to $74,999 $75,000 to $99,999 $100,000 or more Prefer not to answer	4 (9.3%) 6 (14.0%) 8 (18.6%) 16 (37.2%) 9 (20.9%)
Cancer diagnosis Breast Genitourinary Other	38 (88.4%) 2 (4.7%) 3 (7.0%)
Cancer stage Stage I Stage II Stage III Stage IV or metastatic Prefer not to answer	4 (9.3%) 1 (2.3%) 1 (2.3%) 16 (37.2%) 21 (48.8%)
Treatments received Chemotherapy Radiotherapy Surgery Other (e.g., hormonal therapy and immunotherapy)	24 (55.8%) 31 (72.1%) 36 (83.7%) 18 (41.9%)
Menopausal status Pre-menopause Going through menopause Post-menopause	10 (23.3%) 9 (20.9%) 24 (55.8%)
Total weekly physical activity (min)	71.8 (60.0)
Moderate-to-vigorous physical activity per week (min)	31.9 (31.0)
Perceived social support (score range = 1 to 5) Satisfaction with quantity Satisfaction with quality Sources of social support No one Friend with cancer Friend without cancer Family member without cancer People posting online Exercise professional Support group Other (not specified)	2.79 (1.2) 2.86 (1.3) 15 (34.9%) 3 (7.0%) 9 (20.9%) 19 (44.2%) 4 (9.3%) 5 (11.6%) 6 (14.0%) 1 (2.3%)

### Main results

#### Primary outcome

Overall, participants rated 2U Strong above the predetermined benchmark (*M*_score_ > 4.0 out of 5) and were satisfied with 2U Strong (*M*_score_ = 4.7, *SD* = 0.54; *M*_range_ = 2.93 to 5.00). Constructs with the highest mean scores were satisfaction with the security of Zoom (*M*_score_ = 4.9, *SD* = 0.48), convenience of the program (*M*_score_ = 4.8, *SD* = 0.42), usefulness of the exercise sessions (*M*_score_ = 4.8, *SD* = 0.61), information being consistent with information from health care providers (*M*_score_ = 4.8, *SD* = 0.45), and information being from a credible source (*M*_score_ = 4.8, *SD* = 0.49). All satisfaction constructs are summarized in Table 2.

**Table 2:** Summary of the satisfaction with 2U Strong items (*N* = 43 participants)

Item	*M (SD)*
Easy to use	4.6 (.67)
Convenient to use	4.8 (.42)
Enjoyed exercise sessions	4.6 (.81)
Zoom was secure	4.9 (.48)
Satisfied with the exercise sessions	4.7 (.61)
Exercise sessions were acceptable	4.7 (.76)
Exercise sessions were understandable	4.7 (.72)
Exercise sessions were useful	4.8 (.61)
Exercise sessions were enjoyable	4.5 (.82)
Information provided was consistent with information from health care providers	4.8 (.45)
Information was from credible sources	4.8 (.49)
I would use 2U Strong again	4.4 (1.0)
I liked Zoom	4.5 (1.0)

*Note*: Range of scores is from 1 to 5

#### Exploratory outcomes

MVPA significantly increased from T0 (*M*_T0_ = 31.9, *SD* = 31.0) to T1 (*M*_T1_ = 47.3, *SD* = 30.2) to T2 (*M*_T2_ = 40.4, *SD* = 31.6; *F* (2, 34) = 3.5, *p* < 0.05; ${\eta }_{p}^{2}$ = 0.19). Perceived social support quantity increased from T0 (*M*_T0_ = 2.8, *SD* = 1.2) to T1 (*M*_T1_ = 4.2, *SD* = 1.0) to T2 (*M*_T2_ = 3.8, *SD* = 1.1; *F* (2, 34) = 18.6,* p* < 0.001; ${\eta }_{p}^{2}$ = 0.45). Perceived social support quality also significantly increased from T0 (*M*_T0_ = 2.9, *SD* = 1.3) to T1 (*M*_T1_ = 4.1, *SD* = 1.1) to T2 (*M*_T2_ = 3.8, *SD* = 1.3; *F* (2, 34) = 13.9, *p* < 0.001; ${\eta }_{p}^{2}$ = 0.38). Total minutes of physical activity, exercise self-efficacy, barrier self-efficacy, and HRQoL did not significantly increase from T0 to T1 to T2 (${\eta }_{p}^{2}$ range = 0.001 to 0.06).

#### Program feedback

Based on the open-ended responses, participants identified four aspects of 2U Strong that they enjoyed. First, participants consistently reported the importance of having a qualified and empathetic instructor. Participants enjoyed the convenience of virtual sessions and the ability to exercise from home. Participants appreciated the structured and progressive design of the exercise program. Finally, participants reported physical and emotional benefits. Participants did not enjoy three aspects of 2U Strong including issues related to connectivity and audio and video quality of the sessions. Some participants were not satisfied with the level of bonding and interaction within their exercise group. Participants also expressed a desire for more information about specific exercises and longer program duration. Areas of improvement include providing additional exercise sessions, extending the duration of the program, and facilitating discussions and social support. Findings including themes, representative quotes, and frequencies can be found in the Supplemental Files (Supplemental Table 2).

## Discussion

The study aimed to assess participants’ overall satisfaction with the exercise program, qualitatively identify areas of improvement, and determine the preliminary effects of the exercise program on self-reported PA, perceived social support, exercise self-efficacy, barrier self-efficacy, and HRQoL, and to solicit feedback from participants. Participants were very satisfied with the exercise program. The security of Zoom videoconference software and convenience and usefulness of the sessions were rated with the highest satisfaction scores. The exercise program significantly increased self-reported MVPA from T0 (baseline) to T2 (follow-up) and perceived social support quantity and quality with large effect sizes. Exploratory outcomes including total minutes of PA, exercise self-efficacy, barrier self-efficacy, and HRQoL did not significantly increase from T0 to T2.

Participants were satisfied with 2U Strong providing an overall rating that exceeded the predetermined benchmark. Individual items including the information received and usefulness of the exercise sessions higher than the overall mean score and may have contributed to the high rating. In previous studies, cancer survivors have also reported that the convenience of remote-delivered PA positively impacted program acceptability and satisfaction scores [13, 17]. Suggestions for improvement included fostering more interactions within exercise groups. Previous research has suggested opening the videoconference space 15 min early may facilitate within-group interaction [16]. To promote future intervention scaling efforts, inclusion of a process evaluation is necessary to determine which strategies are feasible and most impactful on participant satisfaction, PA levels, and cancer-related outcomes [21].

As expected, there was a significant increase in self-reported MVPA from T0 to T2 among participants enrolled in 2U Strong suggesting an intervention effect. Importantly, the increase in MVPA remained relatively stable from T1 (post-program) to T2 (follow-up). The current findings mirror that of previous research where young breast cancer survivors significantly increased their objectively measured MVPA from baseline to three-months post-intervention follow-up in a remote-delivered peer-support PA intervention with technology support [37]. In a videoconference-delivered exercise program for cancer survivors, aerobic MVPA significantly increased from baseline to post-program among adult participants. However, this sample was more active at the start compared to the participants in the current study [18]. Future research is encouraged to use optimization study designs to determine which strategies are more predictive of post-intervention MVPA [38].

Perceived social support quantity and quality significantly increased from T0 to T2. Effect sizes were large suggesting that 2U Strong had a statistical effect on social support perceptions. The current findings are aligned with previous research where social support and PA adherence were positively associated [39]. Specifically, during a previous 12-week remote-delivered individualized exercise program, participants reported a statistically significant increase in feelings of support (*p* < 0.05) and a decrease in loneliness (*p* < 0.05) [19]. Few previous remote-delivered PA interventions incorporate a “buddy” as a means of increasing social support, despite research suggesting that cancer survivors prefer being assigned an exercise buddy [40]. Importantly, in the current study, social support strategies such as an assigned buddy, meetings with the instructor, and group-based exercise format may have contributed to the positive perceptions of social support throughout the study period.

In contrast, from T0 to T2, there were no significant changes in exercise and barrier self-efficacy and HRQoL and the effect sizes were small. While it is likely that the current study was not sufficiently powered to detect differences in these outcomes, other factors may have also contributed. For example, a previous systematic review suggests that increasing HRQoL may require a longer intervention that is 12 weeks or more [41]. Increasing the sample size and extending the follow-up period may clarify why the current findings lack significance.

This study is not without its limitations. Causal inferences cannot be drawn given the use of a single-arm study design that is also statistically limited by the small sample size. The study design was selected for pragmatism given the community-based approach of the current research [42]. PA was self-reported, which may have induced self-report bias [43]. Future research should consider objective measures for physical activity levels such as accelerometer-derived PA to reduce bias. The sample was predominantly non-Hispanic White with bachelor’s degrees or higher, and had household incomes of ≥ $100,000; thus, the findings have limited generalizability as the sample is not representative of women diagnosed with cancer in the USA. Given the disparities in cancer mortality rates and adherence to cancer-specific PA guidelines, additional research is needed among racially/ethnically and socioeconomically diverse cancer populations to identify relevant intervention and implementation strategies [44].

The findings demonstrated that 2U Strong was rated as satisfactory and improved MVPA and perceived social support. However, other exploratory outcomes did not change. Based on the promising findings, future hybrid implementation-effectiveness studies with larger and more-diverse populations are needed to examine the impact of 2U Strong on self-reported cancer-related outcome.

## Supplementary Information

Below is the link to the electronic supplementary material.

Supplementary file1 (DOCX 29 KB)

## Data Availability

No datasets were generated or analysed during the current study.
