# Supplementary material for: Evaluation of a community-based remotely delivered exercise program (2Unstoppable Strong) for women diagnosed with cancer
Source: Support Care Cancer. 2025 Jul 25;33(8):721. doi: 10.1007/s00520-025-09772-4 (PMC12296796; doi:10.1007/s00520-025-09772-4)
Supplement: Supplementary file 1 — Supplementary file1 (DOCX 29 KB) [file 520_2025_9772_MOESM1_ESM.docx]

**Supplemental Table 1**

*Overview of 2U Strong Exercise Structure and Design*

| Week | Focus | Description |
| --- | --- | --- |
| 10-week Program for Cancer Survivors with Metastatic Disease | | |
| 1 | Introduction to Exercises | - Range of motion exercises - Compound strength exercises - Cardio movements |
| 2 | Cardiovascular Endurance | - Cardio exercises - Balance exercises (>5 minutes) - Endurance testing |
| 3 | Strength & Endurance Building | - Extended cardio sessions (increased durations and intensity) - Bodyweight exercises - Range of motion work - Balance exercises (continued practice and improvement) |
| 4 | Strength, Agility, & Flexibility | - Resistance training (adding weights and resistance bands) - Single-leg balance exercises - Increased stretching time |
| 5 | Increased Intensity | - Advanced cardio and strength exercises (higher intensity and complexity) - Monitoring improvements (tracking endurance, strength, and energy levels) |
| 6 | Peak Performance | - Challenging all previous exercises (endurance and strength) - Increased pace and intensity - Form refinement |
| 7 | Muscle Isolations | - Isolation exercises targeting major muscle groups - Use of bodyweight, weights, and resistance bands - Extended balance and flexibility time |
| 8 | Cardio Focus | - Intensive cardio workout - Heart rate monitoring (tracking cardio endurance improvements) |
| 9 | Integrated Cardio & Strength | - Combined cardio and strength exercises - Full body engagement |
| 10 | Accomplishment Celebration | - Recognize improvements in endurance, strength, and overall fitness |
| 6-week Program for Cancer Survivors with Metastatic Disease | | |
| 1 | Ease into Exercise | - - Basic stretches (targeting major muscle groups: arms, legs, hips) - Range of motion exercises |
| 2 | Flexibility & Strength | - - Flexibility exercises (emphasis on lengthening muscles)   - Strengthening movements (using household objects: bath towel, pillow) - Breath-focused exercises (ensuring proper movement and muscle engagement) |
| 3 | Adding Resistance | - - Resistance band exercises   - Continued flexibility work - Balance and core exercises |
| 4 | Strength, Agility, & Flexibility | - - Blended progressive exercises (combining strength, agility, and flexibility) - Enhanced mobility routines |
| 5 | Increased Energy & Cardio | - - Cardio exercises - Building on previous weeks (combining strength and flexibility) |
| 6 | Finishing Strong | - Recognize improvements in strength and flexibility |

***Supplemental Table 2***

*2US Program Evaluation Themes*

| Theme | Frequency *n*(%) | Representative Quotes (Participant Code) |
| --- | --- | --- |
| Enjoyable Aspects | | |
| Importance of instructor | 17(42%) | "[Exercise Instructor 1] and [Exercise Instructor 2] are amazing and supportive." (2US_110)  “[Exercise Instructor 1] was easily approachable for clarification/problem solving with exercises.” (2US_131)  [I liked] “The enthusiasm and accepting nature of the instructor.” (2US_111) |
| Convenience of sessions | 15(36%) | “Being provided equipment that you can use at home, free to Cancer patients, exercises were doable even with limitations.” (2US_614)  “Convenience of doing it from home, support of the group, instructor.” (2US_126)  [I liked the] “Ease of use, materials provided.” (2US_123) |
| Structure and design of exercise program | 14(34%) | “The structured program with progressive exercise (each week built on the last), gave me accountability to show up each week.” (2US_109)  “The incremental nature of difficulty of the classes (instilled confidence) and addresses specific issues related to cancer recovery - i.e. lymphedema.” (2US_111) |
| Emotional and physical benefits | 24(58%) | “It just feels different and better exercising with people who have experienced cancer - a way to connect via an activity.”  (2US_130)  “I love the fact that it helped me to get back in the swing of exercising and helped encouraged my walking routine. This program has also helped me to encourage others to try it and start their own exercise routines.” (2US_613)  “I liked the comradery, friendship, and sharing laughs.” (2US_139) |
| Aspects Participants Did Not Enjoy | | |
| Connectivity and audio issues | 6(17%) | “I did not like the technical issues.”  2US_129  “I did not like the limitations with Zoom, like the music being hard to hear and group members being hard to see.” (2US_107) |
| Lack of satisfaction with interaction within the exercise group | 11(31%) | "Our group didn't really bond” (2US_108)  “The group didn't 'gel'—not enough time to talk and form relationships." (2US_112)  “My buddy pairing was incompatible.”  (2US_138) |
| Desire for additional information and longer duration of program | 9(26%) | "I would like more information about why it is so important to exercise."  (2US_607)  "Wish the program was more weeks and would like a class that follows this one for MBC [metastatic breast cancer]." (2US_615)  "I wish the program was longer” (2US_131,  2US_602) |
| Suggestions for Improvement | | |
| Additional resources | 15(47%) | " Provide an instructional handout for each of the new exercises for each class." (2US_131)  “One day, I would love to access the classes as an App on TV (i.e. Essentrics).” (2US_111) |
| Extending duration of the program | 9(28%) | "I wish the program was longer, 12 weeks"(2US_602)  “Wish the program was more weeks and would like a class that follows this one for MBC.” (2US_615) |
| Social interaction facilitation | 8(25%) | “Our group didn't really bond” 2US_108  “There was not enough socializing with other classmates.” (2US_115) |
